# A Mobile App to Facilitate Socially Distanced Hospital Communication During COVID-19: Implementation Experience

**DOI:** 10.2196/24452

**Published:** 2021-02-23

**Authors:** Emeka C Anyanwu, R Parker Ward, Atman Shah, Vineet Arora, Craig A Umscheid

**Affiliations:** 1 Section of Cardiology University of Chicago Chicago, IL United States; 2 Section of General Internal Medicine University of Chicago Chicago, IL United States; 3 Center for Healthcare Delivery Science and Innovation University of Chicago Chicago, IL United States

**Keywords:** adoption, communication, COVID-19, hospital, inpatient, mHealth, mobile app, telemedicine

## Abstract

**Background:**

COVID-19 has significantly altered health care delivery, requiring clinicians and hospitals to adapt to rapidly changing hospital policies and social distancing guidelines. At our large academic medical center, clinicians reported that existing information on distribution channels, including emails and hospital intranet posts, was inadequate to keep everyone abreast with these changes. To address these challenges, we adapted a mobile app developed in-house to communicate critical changes in hospital policies and enable direct telephonic communication between clinical team members and hospitalized patients, to support social distancing guidelines and remote rounding.

**Objective:**

This study aimed to describe the unique benefits and challenges of adapting an app developed in-house to facilitate communication and remote rounding during COVID-19.

**Methods:**

We adapted moblMD, a mobile app available on the iOS and Android platforms. In conjunction with our Hospital Incident Command System, resident advisory council, and health system innovation center, we identified critical, time-sensitive policies for app usage. A shared collaborative document was used to align app-based communication with more traditional communication channels. To minimize synchronization efforts, we particularly focused on high-yield policies, and the time of last review and the corresponding reviewer were noted for each protocol. To facilitate social distancing and remote patient rounding, the app was also populated with a searchable directory of numbers to patient bedside phones and hospital locations. We monitored anonymized user activity from February 1 to July 31, 2020.

**Results:**

On its first release, 1104 clinicians downloaded moblMD during the observation period, of which 46% (n=508) of downloads occurred within 72 hours of initial release. COVID-19 policies in the app were reviewed most commonly during the first week (801 views). Users made sustained use of hospital phone dialing features, including weekly peaks of 2242 phone number dials, 1874 directory searches, and 277 patient room phone number searches through the last 2 weeks of the observation period. Furthermore, clinicians submitted 56 content- and phone number–related suggestions through moblMD.

**Conclusions:**

We rapidly developed and deployed a communication-focused mobile app early during COVID-19, which has demonstrated initial and sustained value among clinicians in communicating with in-patients and each other during social distancing. Our internal innovation benefited from our team’s familiarity with institutional structures, short feedback loops, limited security and privacy implications, and a path toward sustainability provided by our innovation center. Challenges in content management were overcome through synchronization efforts and timestamping review. As COVID-19 continues to alter health care delivery, user activity metrics suggest that our solution will remain important in our efforts to continue providing safe and up-to-date clinical care.

## Introduction

COVID-19 has fundamentally altered health care delivery. Hospitals have rapidly established and revised protocols to promote optimal patient care with minimized contact [1], particularly considering the limited supplies of personal protective equipment (PPE) [2]. Similarly, clinicians have altered the most basic aspects of patient care owing to social distancing while simultaneously adjusting to near daily changes in practice protocols. In this environment, the use of telehealth has increased substantially [3-5], bolstered by a temporary relaxation of technology requirements [6].

Owing to challenges in disseminating rapidly changing policies via email and the hospital intranet to a newly remote workforce, we adapted an existing mobile app to improve information accessibility at our institution. This app allows for direct dialing to in-patient rooms and facilitates clinician-patient communication while simultaneously minimizing contact and PPE use. This study describes the implementation and use of this app during the early stages of COVID-19 at our academic medical center as well as the benefits and challenges associated with its use.

## Methods

At the onset of COVID-19, the Hospital Incident Command System (HICS) was established at the University of Chicago Medical Center and began distributing institutional policies and guidelines via email and the hospital intranet. The HICS soon determined that mobile communication might help overcome the limitations of communicating via email and the intranet, which are encountered by many frontline workers, but it was infeasible to develop a new mobile app owing to the overwhelming demands of the pandemic on the information technology (IT) team. The leadership began considering how existing communication technologies could be adapted rapidly to meet these demands.

The mobile app moblMD [7] (Multimedia Appendix 1) was initially implemented at the University of Chicago Medical Center in April 2018 as part of a feasibility study. The app provided a hospital directory, helped dial hospital phone numbers, and distributed institutional guidelines among users. Developed in-house by a cardiology fellow, it was a project of the Resident Advisory Council and has been used by 156 in-house staff since before COVID-19.

Instead of developing a new app, we rapidly adapted moblMD to (1) distribute COVID-19–related policies from the HICS team and (2) help clinicians search for and dial patient bedside phone numbers, as well as charge nurse and unit secretary phones, in order to promote social distancing and remote patient rounding.

Members of the HICS team were individuals playing different roles including nursing, strategy, and communication; they were selected to validate app content. To coordinate messaging, shared documents were used to ensure that the distribution of updates via email and the intranet were reflected in the content of moblMD. This small group of test users were provided preview access to updates before each release. After a rapid privacy and IT security review, instructions were distributed to all attending physicians, in-house staff, and advanced practitioners on March 29, 2020, and to all nurses on March 30, 2020. A new class of in-house staff was selected on June 22, 2020, and a new version of the app with a more intuitive interface for dialing patient rooms was developed from June 25, 2020. Accordingly, a reminder announcement was distributed on July 14, 2020.

**Figure 1:**
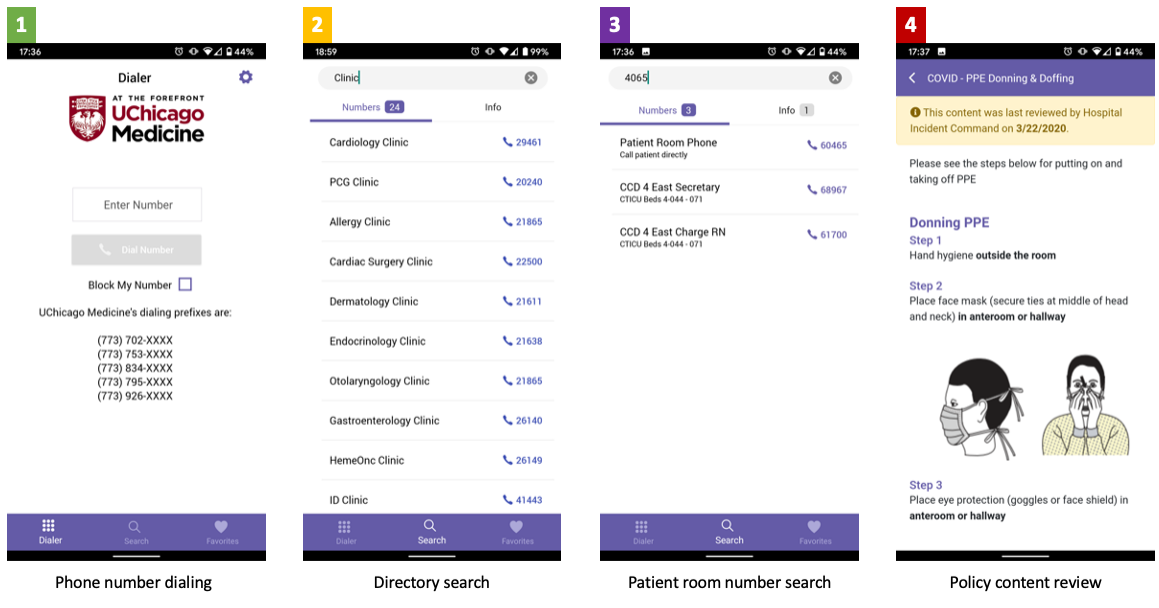
Representative screenshots of user activity categories within the moblMD app, ranked in descending order of usage.

Anonymized aggregate user activity data from the app server were reviewed approximately 2 months before and 4 months after the initial announcement (February 1 to July 31, 2020). Outcomes included the number of app users and user actions, the latter categorized as follows: general phone directory search, patient room phone number search, phone number dialing, and policy content review (Figure 1). Policy content reviews were examined for both frequency and page views. The institutional review board approved the use of moblMD (IRB18-0082).

## Results

**Figure 2:**
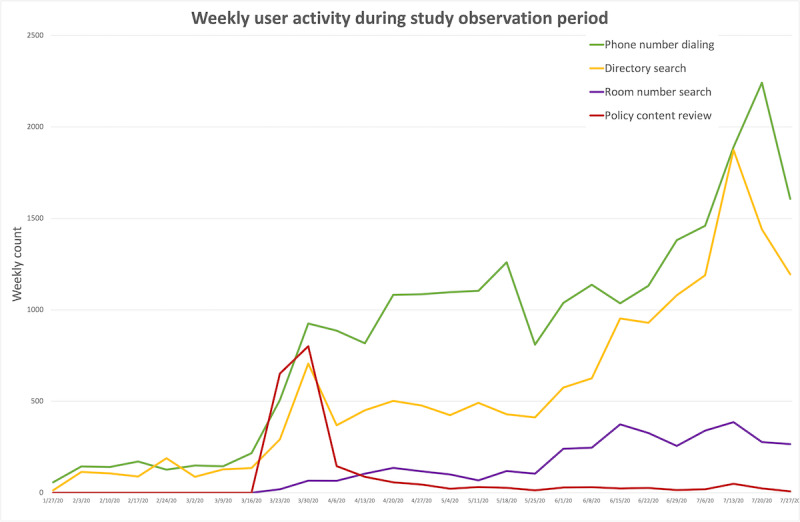
Aggregate app user activity over the study period (February 1 to July 31, 2020). Note: The policy content review and room number search functions were not implemented until March 14, 2020. The last charted week was not a full week.

Within 4 months of release, 1004 unique users downloaded moblMD, with 46% (n=508) downloads within 72 hours of the first announcement, and 10% (n=110) downloads within 72 hours of the second announcement (Multimedia Appendix 2) [8,9]. Weekly total numbers of user app actions are shown in Figure 2.

Within 72 hours of the first announcement, policy content was accessed at a similar frequency to that of directory searches and direct dialing, peaking at 801 weekly views. The most viewed policy contents during the study period were COVID-19 Important Contacts (437 views); COVID-19 Frequently Asked Questions (410 views); and COVID-19 Testing, patients under investigation, and Exposure (253 views) (Multimedia Appendix 3). In subsequent months, moblMD was used most frequently for hospital phone number dialing, including weekly peaks of 2242 phone number dials, 1874 directory searches, and 277 patient room phone number searches through the last 2 weeks of the observation period. Users submitted 56 content-related suggestions through the app during the observation period and many others through informal channels. The overall process for app implementation and timelines are illustrated in Multimedia Appendix 4.

## Discussion

### Principal Findings

During the early stages of COVID-19, we found that our in-house designed mobile app moblMD provided clinicians access to rapidly evolving institutional policies and protocols, facilitated remote patient care, and gained widespread durable use at our large academic medical center.

The most immediate impact of our intervention was to provide mobile access to new and changing hospital protocols in response to COVID-19. Prior to this, frequent communication regarding rapidly changing policies from our HICS team were only accessible via a series of emails and the intranet. These channels could be particularly ineffective and overwhelming for clinicians redeployed to new clinical roles [10]. While a mobile format improved information accessibility for frontline clinicians, it was challenging to keep disparate information sources synchronized. Aligning of moblMD content with traditional communication channels, focusing on high-impact policies on patient care (eg, PPE use, intensive care unit guidelines, etc), and labeling entries with the time of last review and the corresponding reviewer (Figure 1, box 4) helped address these challenges. Another challenge in managing app content was curating full-length content in .docx and PDF formats for brevity and mobile-friendly formatting. In a subsequent update, a toggle was enabled to link PDF documents. As expected, content views, such as instructions for PPE use and exposure protocols, peaked within 72 hours of the first announcement of the app as clinicians first consumed content, and such content was less likely to require repeated views.

The most durable impact of moblMD was the facilitation of remote patient care. Before COVID-19, our clinicians, like many others [11], routinely used their smartphones at the point of care. Although patient bedside phone numbers were previously accessible via the hospital call center or within the electronic health record, our mobile solution enhanced communication efficiency by providing a faster alternative. By facilitating mobile communication, we reduced the need for in-person communication as remote patient care became the norm to minimize PPE use and clinician exposure.

Many institutions have implemented other forms of in-patient telehealth or electronic PPE through which patients have video calls with clinicians through hospital-owned devices [12-14]. While this is a viable solution, it is costlier than in-person consultations and cannot be rapidly implemented at most institutions. Our intervention allowed for similar remote patient care in a matter of days, and clinicians had complete access to remote patient care through their smartphones.

Concurrent with previous reports, certain benefits and unique challenges are associated with internally sourced innovations [15]. Having been developed in-house, moblMD benefited from our team’s understanding of the institutional culture and structure, which resulted in shorter feedback loops for content and feature updates. Feedback from the HICS team was delivered rapidly to the developer, and the app also included a feedback feature allowing clinicians to request updates. Clinicians were quick to suggest phone numbers relevant to their practice areas. Additionally, early user feedback prompted an interface update to further facilitate patient room search. Finally, although the app was largely known to the users, its distribution on the Google Play Store and the Apple App Store was based on app reviews, which took several days to obtain and needed to be factored in to discussions on feature requests.

Mobile communication and app use in health care settings has led to concerns regarding patient privacy [16] and information security [17-19]. Prior to release, an internal security audit and IT review were conducted in 5 business days despite this process typically taking much longer in general. moblMD was granted security approval expeditiously because it did not interface with the hospital infrastructure or collect user information other than an email address used for authentication. As a one-way communication channel, the risk of inappropriate transmission of patient information was limited. Following approval, the security team recommended follow-up evaluation after COVID-19 to address noncritical concerns.

Furthermore, it is important to address support and sustainability in our rollout of moblMD. Fortuitously, our Center for Healthcare Delivery Science and Innovation had recently announced an internal funding opportunity for COVID-19 innovations that provided a critical path towards sustainability. This helped advocate for our innovation within the hospital leadership, financially supported app infrastructure, and provided personnel with time to update the app content. Based on our experience, the Center for Healthcare Delivery Science and Innovation has adopted an innovation intake process to connect internal innovators with funding and resources in the IT, compliance, and legal sectors to facilitate early growth and validation [20].

### Conclusion

We successfully adapted a mobile app to promptly facilitate remote patient care and disseminate COVID-19–related hospital protocols. Our mobile solution scaled without issue following announcements to thousands of users. The team’s familiarity with institutional structures, short feedback loops, limited security and privacy implications, and a path toward sustainability provided by our innovation center were the key determinants to the successful implementation of our app. Challenges in content management were overcome through synchronization efforts and timestamping review. As COVID-19 continues to alter health care delivery, user activity metrics suggest that our solution will remain important in our efforts to continue providing safe and up-to-date clinical care.

## Appendix

Multimedia Appendix 1 Video demonstration of the basic functionality of the moblMD app.

Multimedia Appendix 2 Growth of moblMD app user accounts over time. Time markers highlighting the date of the first COVID-19–related death, the start of the stay-at-home order in Illinois, and our hospital-wide app announcement.

Multimedia Appendix 3 Policy content view counts during the study period (February 1 to July 31, 2020). Frequently asked questions included those regarding employee support resources, COVID-19 support clinics, blood donation/research, and donations of personal protective equipment.

Multimedia Appendix 4 Overview of the app implementation process and timeline.

## Abbreviations

HICS: Hospital Incident Command System
IT: information technology
PPE: personal protective equipment
