# Supplementary figures and images for: A Mobile App to Facilitate Socially Distanced Hospital Communication During COVID-19: Implementation Experience
Source: JMIR Mhealth Uhealth. 2021 Feb 23;9(2):e24452. doi: 10.2196/24452 (PMC7903979; doi:10.2196/24452)

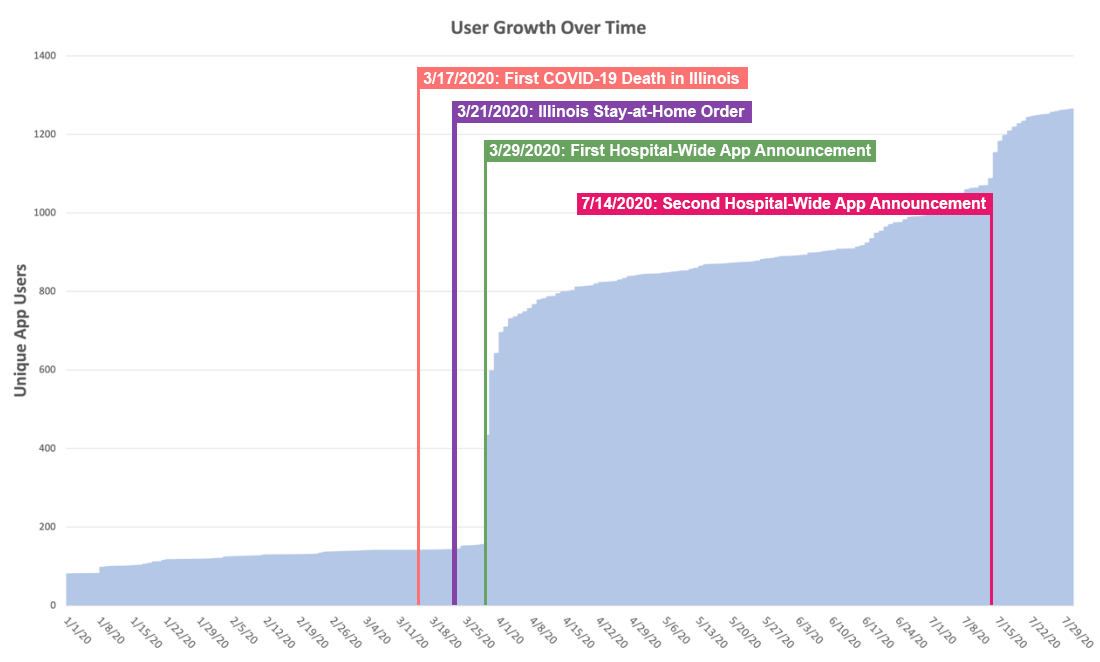

Supplement: Multimedia Appendix 2 [file mhealth_v9i2e24452_app2.png]

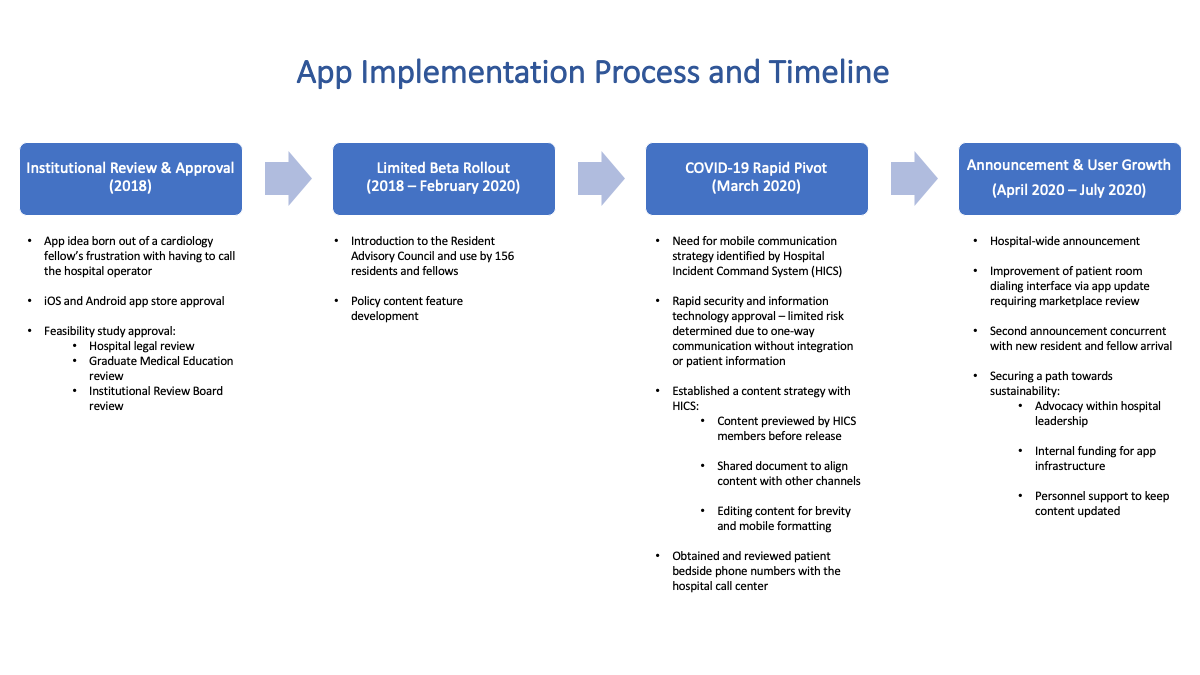

Supplement: Multimedia Appendix 4 [file mhealth_v9i2e24452_app4.png]
