# Supplementary material for: A Mobile App to Facilitate Socially Distanced Hospital Communication During COVID-19: Implementation Experience
Source: JMIR Mhealth Uhealth. 2021 Feb 23;9(2):e24452. doi: 10.2196/24452 (PMC7903979; doi:10.2196/24452)
Supplement: Multimedia Appendix 3 [file mhealth_v9i2e24452_app3.docx]

| Content Page | View Count |
| --- | --- |
| COVID - Important Contacts | 437 |
| COVID - Frequently Asked Questions^1^ | 414 |
| COVID - Testing, PUIs, Exposure | 253 |
| COVID - ECMO & Mechanical Support | 203 |
| COVID - Admissions, Ambulatory, and Other Encounters | 193 |
| COVID - Employee Furlough & Universal Masking | 173 |
| COVID - Isolation & PPE Protocols | 164 |
| COVID - PPE Donning/Doffing Instructions | 157 |

**Appendix Table 1** – Policy content view counts during the study period (February 1 – July 31, 2020) ^1^Frequently asked questions included those regarding: employee support resources, COVID-19 support clinics, blood donation/research, and PPE donations.
